# Vehicle emissions and consumer information in car advertisements

**DOI:** 10.1186/1476-069X-7-14

**Published:** 2008-04-29

**Authors:** Nick Wilson, Anthony Maher, George Thomson, Michael Keall

**Affiliations:** 1 Department of Public Health, University of Otago, Wellington, New Zealand

## Abstract

**Background:**

The advertising of vehicles has been studied from a safety perspective but not in terms of vehicle air pollutants. We aimed to examine the content and trends of greenhouse gas emissions and air pollution-related information, in light passenger vehicle advertisements.

**Methods:**

Content analysis of the two most popular current affairs magazines in New Zealand for the five year period 2001–2005 was undertaken (n = 514 advertisements). This was supplemented with vehicle data from official websites.

**Results:**

The advertisements studied provided some information on fuel type (52%), and engine size (39%); but hardly any provided information on fuel efficiency (3%), or emissions (4%). Over the five-year period the reported engine size increased significantly, while fuel efficiency did not improve.

For the vehicles advertised, for which relevant official website data could be obtained, the average "greenhouse rating" for carbon dioxide (CO_2_) emissions was 5.1, with a range from 0.5 to 8.5 (on a scale with 10 being the best and 0.5 being the most polluting). The average CO_2 _emissions were 50% higher than the average for cars made by European manufacturers. The average "air pollution" rating for the advertised vehicles was 5.4 (on the same 1–10 scale). The yearly averages for the "greenhouse" or "air pollution" ratings did not change significantly over the five-year period. One advertised hybrid vehicle had a fuel consumption that was under half the average (4.4 versus 9.9 L/100 km), as well as the best "greenhouse" and "air pollution" ratings.

**Conclusion:**

To enhance informed consumer choice and to control greenhouse gas and air pollution emissions, governments should introduce regulations on the content of vehicle advertisements and marketing (as started by the European Union). Similar regulations are already in place for the marketing of many other consumer products.

## Background

The advertising of vehicles has been studied previously, due to concerns that it may adversely influence safety-related behaviours [1-5]. However, there are no published Medline-indexed articles where such advertising has been analysed in terms of greenhouse gas emissions or other air pollutant emissions. This is despite the growing international concern around both these types of emissions, given the current and potential health impacts of climate change, and from the direct health effects of vehicle emissions [6,7]. To determine the current situation in this area of marketing, we undertook a content analysis of advertisements for light passenger vehicles.

## Methods

New Zealand was selected as the country for the data collection, given that the authors reside in this country and hence were most familiar with the New Zealand specific data sources and the contextual issues of this country's car advertising market.

### Magazine selection and search strategy

As detailed elsewhere in a separate road safety analysis using the same sampling frame [8], the two highest circulation monthly current affairs/lifestyle magazines specific to New Zealand were selected (ie, *Metro *and *North & South*). These magazines have a reported readership of 126,000 and 268,000 respectively according to their publisher (ACPMEDIA) with *North & South *having a national focus and *Metro *having a focus on the country's largest city of Auckland. All the issues for the five-year period from 2001 to 2005 were hand-searched on a page-by-page basis, and if the vehicle advertisement took up more than a quarter of the page (an arbitrary cut-off), then it was included in the study and photocopied. Only the advertisements that dealt with a specific model of car, sports utility vehicle (SUV) or similar light vehicle (primarily for passengers, with fewer than eight seats), were considered.

### Data for the content analysis

Data were collected from the advertisements on: the make, whether the vehicle was a SUV, whether the vehicle was "four wheel drive" or "all wheel drive" (4WD/AWD), engine size, type of fuel (unless mentioned it was assumed to be petrol), fuel efficiency and emissions profile (if reported) or any features related to these. Vehicles were classified as SUVs if they were specifically defined in the advertisement as being a SUV, recreational vehicle (RV), or all-terrain passenger vehicle, or if they were defined as a SUV on vehicle manufacturers' websites.

For each vehicle model found in the advertisements (determined by different vehicle company, make and engine size), additional data for fuel efficiency were sourced using an official government website [9]. Fuel efficiency was considered, as poorer efficiency is directly associated with greenhouse gases per kilometre travelled, and can be related to increased emissions of certain air pollutants (though this is not always the case as further considered in the Discussion Section). Emissions data (in the form of "greenhouse" and "air pollution" ratings) for each different vehicle were also sourced where possible using an official Australian website [10], as there was no suitable local (New Zealand) equivalent. The basis for these two ratings are detailed elsewhere [10], but the "greenhouse rating" is based on the CO_2 _emissions of the vehicle, while the "air pollution" rating is based on the emissions of carbon monoxide (CO), hydrocarbons (HC) and oxides of nitrogen (NO_x_) (and diesel vehicles must also meet a limit for the emission of particulate matter (PM)). The best "greenhouse rating" of 10 is for the lowest rate of CO_2 _emissions of <= 60 g/km. The same test conditions are used for determining air pollutant emissions, CO_2 _emissions and fuel consumption and are based on the internationally recognised United Nations ECE Regulations (ECE R83 and ECE R101). These are the same test conditions used for New Zealand fuel efficiency calculations, and in European testing.

Each vehicle website detailed in the advertisements was collected and accessed. From these websites, fuel efficiency and emissions data were collected.

### Data collation and analysis

Data were analysed using EpiInfo (CDC, Atlanta). To best approximate the advertisement impact on consumers exposed to the magazines, the unit of analysis was a particular advertisement in a particular issue (ie, this meant a larger contribution in the results from advertisements that were repeated in subsequent issues or appeared in both magazines). To assess how the advertised cars compared with cars actually sold in the New Zealand market, we also undertook a comparison for the year 2005 using the top ten selling models of new cars [11]. But since for each model there were many different sized cars, we used the fuel efficiency and air pollution values based on the vehicle that had the median fuel efficiency within the range for that model (using the same data sources as for the advertised vehicles).

### Validation study

As only a single person (AM) classified the advertisement content, a validation study using another person was conducted (see the acknowledgements). It included a 5% random sample of all advertisements (n = 26), and a 15% random sample of those advertisements classified by AM as portraying speed imagery (n = 21) (as part of another study [8]). The inter-rater reliability was at least 94% for key variables relating to this analysis (ie, SUV (94%), 4WD/AWD (94%), fuel efficiency (96%), and emissions data/quotes (96%)).

## Results

### Overall advertising patterns

A total of 514 relevant advertisements were identified for the five-year period (n = 279 for *Metro*, n = 235 for *North & South*) (Table 1). This was made up by a total of 149 different vehicles (determined by vehicle model and engine specifications).

**Table 1:** Advertisements and type of vehicle by year from the magazines surveyed (2001–2005)

**Year**	**SUV**^a^		**Other 4WD & AWD**^b^		**Other vehicles**	
			
	**%**^c^	**N**	**%**^c^	**N**	**%**^c^	**N**
2001	19.1	13	19.1	13	61.8	42
2002	11.5	7	21.3	13	67.2	41
2003	29.5	31	12.4	13	58.1	61
2004	27.6	45	14.7	24	57.7	94
2005	16.2	19	8.5	10	75.2	88

Total	22.4	115	14.2	73	63.4	326

^a ^Vehicles were classified as SUVs if they were specifically defined in the advertisement as being a SUV, recreational vehicle (RV), or all-terrain passenger vehicle, or if they were defined as a SUV on vehicle manufacturers' websites.

^b ^The category "Other 4WD and AWD" included all relevant vehicles where the advertisements mention they are either 4WD or AWD capable (and excluded all SUVs as defined above).

^c ^Percentages in this table reflect the composition *within *years (ie, across the row).

### Emissions and fuel efficiency aspects

Of the vehicles advertised, 22% were SUVs and another 14% were 4WD/AWD vehicles. The advertisements for SUVs and for 4WD/AWDs combined peaked in 2003 and 2004 respectively (Table 1). There was a sharp reduction in the proportion of these vehicles being advertised in 2005 relative to 2004 (eg, for SUVs the rate ratio was 0.59, 95% confidence interval: 0.36, 0.95).

Overall, 53% of advertisements supplied some indication of the type of engine fuel. Of all advertisements, the fuel type mentioned was: petrol (46%), diesel (3%), both petrol/diesel (2%), liquid petroleum gas (LPG) (0.6%), and hybrid (electric/petrol) (0.4%) (Table 2). There was a general decrease in advertisements not specifying the engine fuel type, from 59% in 2001 to 44% in 2005.

**Table 2:** Type of engine fuel mentioned in the vehicle advertisements (n = 514) by year (2001–2005)

**Year**	**Petrol**^a^		**Petrol or diesel**^b^		**Diesel**		**Electric/petrol (hybrid)**		**LPG**		**No fuel information given**^c^	
						
	**%**	**N**	**%**	**N**	**%**	**N**	**%**	**N**	**%**	**N**	**%**	**N**
2001	25.0	17	7.4	5	5.9	4	0	0	2.9	2	58.8	40
2002	52.5	32	0.0	0	0.0	0	0	0	0	0	47.5	29
2003	50.5	53	1.0	1	4.8	5	0	0	0	0	43.8	46
2004	49.1	80	0.6	1	1.2	2	1.2	2	0	0	47.9	78
2005	47.9	56	2.6	3	5.1	6	0.9	1	0	0	43.6	51

Total	46.3	238	1.9	10	3.3	17	0.6	3	0.4	2	47.5	244

^a ^Advertisements with engine information were assumed to be petrol unless otherwise stated.

^b ^Advertisements of vehicles with both petrol and diesel options.

^c ^Advertisements with no information about fuel type or engine specifications.

Only 39% of advertisements gave specific engine capacity values (Table 3). The average engine capacity across all these advertisements was 2.74 litres (range 1.4 to 5.7 L). The lowest average engine capacity for vehicles in the advertisements for each year was 2.37 L in 2002, and this increased each year over the next three years, with this pattern being statistically significant (p = 0.014 on Kruskal-Wallis test).

**Table 3:** Engine size, fuel efficiency and emissions data for advertised vehicles by year and magazine (2001–2005)

**Year/magazine**	**Engine size**^a^		**Fuel efficiency**^b c^		**Greenhouse rating**^b d^		**Air pollution**^b d^	
				
	**Mean capacity (L)**	**N**	**(L/100 km)**	**N**	**Mean rating**	**N**	**Mean rating**	**N**
2001	2.51	23	9.9	66	5.1	43	5.5	45
2002	2.37	25	9.3	59	5.5	39	5.5	39
2003	2.76	37	10.0	103	5.0	77	5.1	77
2004	2.77	64	10.1	161	5.0	124	5.5	124
2005	2.98	51	9.8	117	5.3	99	5.4	99
*Metro*	2.64	98	9.8	277	5.2	213	5.4	214
*North & South*	2.84	102	10.1	229	5.0	169	5.3	170

Total	2.74	200	9.9	506	5.1	382	5.4	384

^a ^"Engine size" taken from actual advertisements.

^b ^Data collection was attempted for all different vehicles (n = 149) and when available it was then extrapolated to the advertisements shown in the magazines.

^c ^"Fuel efficiency" for the advertised vehicles was obtained from an official New Zealand website [9].

^d ^"Greenhouse" and "Air Pollution" ratings are on a scale from 0.5 (worst, most polluting) to 10 (best) based on their CO_2 _emissions and other air pollutant emissions. These were obtained from an official Australian website [10].

Only 3% of advertisements gave specific values for fuel efficiency (either L/100 km or km per tank of fuel), with the average fuel efficiency value of this selection of advertised cars being 5.7 L/100 km (Table 3). Few (4%) made reference to having improved fuel efficiency in the advertised vehicle and only 4% made reference to having reduced emissions or included specific carbon dioxide (CO_2_) data, specific features, or specific guidelines.

In contrast, where provided on the official website [9], the average fuel efficiency for the advertised vehicles was 9.9 L/100 km, and this ranged from 4.4 to 18.6 L/100 km (Table 3). The fuel efficiency did not change significantly over the five-year period.

For the advertised vehicles for which data could be obtained from official websites, the average "greenhouse rating" was 5.1 (Table 3). This ranged from 0.5 to 8.5 (on a scale for CO_2 _emissions, with 10 being the best and 0.5 being the most polluting). The average "air pollution rating" for the advertised vehicles (for which data could be obtained) was 5.4, and this ranged from 0.5 to 8.5. This was on a scale for the level of air pollutant emissions allowable under the Australian standard to which the particular vehicle has been successfully tested for. The yearly averages for the "greenhouse" or "air pollution" ratings did not change significantly over the five-year period. One advertised vehicle (a hybrid) had the lowest fuel consumption (under half the average), and the best "greenhouse" and "air pollution" ratings.

For the 2005 year the values for advertised cars were compared with the top ten selling new car models. These top selling cars were fairly similar to the advertised cars, albeit with slightly smaller engines (2.80 vs 2.98 litres), slightly poorer fuel efficiency (10.0 vs 9.8 L/100 km), slightly better "greenhouse ratings" (5.4 vs 5.3) and slightly better "air pollution ratings" (5.6 vs 5.4).

### Vehicle company websites

Although 89% of advertisements had websites listed, there were only a total of 29 separate vehicle manufacturers and websites involved. Most of these websites provided fuel efficiency data for the advertised vehicles (66%) and 41% provided emissions information (but often using vague terms such as meeting "emissions criteria").

## Discussion

### Interpretation of the major results

The advertisements were poor in supplying much of the basic data on which consumers could make informed vehicle purchase decisions (eg, fuel type, engine size, engine efficiency and emissions). There was also little advertising for vehicles using fuels associated with lower greenhouse gas and certain other exhaust emissions per distance travelled (ie, diesel, LPG and electric/petrol mixes). Even vehicle manufacturer websites were poor at supplying data about fuel efficiency and, in particular, emissions information. This is despite having in-depth detail about engine specifications and performance data.

Given that much of the use of light passenger vehicles in developed countries (including New Zealand [12]) is for only one or two passengers, the advertising of vehicles using as much as 18.6 litres per 100 km is counter to the achievement of achieving national and international climate change and energy security goals. The significant increase in average engine size, where the size was given in the advertisements, may also be problematic for this reason.

The average "greenhouse rating" was 5.1, where a score of five equates to an average CO_2 _emission level of 241–260 g/km [10]. In comparison, the average for cars made by European manufacturers in 2006 was 160 g/km [13] (using the same testing procedure). Furthermore, the European Commission has a proposed target of 130 g/km (for all new cars sold by 2012) [14], and still much lower levels are possible with current technologies (eg, 102 g/km for the *BlueMotion Polo *[15]). Therefore the vehicles advertised in this study appear to be, on average, far more polluting than current European cars, and considerably more polluting than the proposed new European target. This again suggests that the advertising of vehicles with poor greenhouse and air pollution ratings (of as low as 0.5) appears to be counter to the achievement of the national climate change and air quality goals.

Where found in official websites, the average "air pollution rating" of the vehicles advertised was 5.4 out of 10 (with 10 being best or least polluting). While being slightly better than the current Australian emission standard for petrol-fuelled vehicles of 5, the 5.4 average for this New Zealand setting falls just short the standard for new vehicles in Europe from 2005 [10].

The general comments made above assume that improved fuel efficiency and use of smaller and diesel-powered vehicles are related to lower greenhouse gas emissions and also to lower levels of other harmful vehicle emissions. But caveats to this general pattern need to be considered:

• Sometimes larger vehicles are the most efficient for achieving certain tasks (ie, for moving larger numbers of people and for certain occupational groups such as trades workers and farmers). Indeed, if a petrol-engine vehicle is "underpowered" (for the load transported) then it is more likely to go into "enrichment mode" that produces higher carbon monoxide (CO) levels [16].

• Some larger vehicles may have compensatory features that partly ameliorate their lower fuel efficiency relative to smaller cars (eg, better transmission management, cylinder deactivation, and improved valve timing strategies).

• Comparisons of emissions from petrol, diesel and other engines are complex [17,18]. Diesel engines are generally more efficient than petrol engines (they have a higher compression ratio, and diesel fuel also contains more energy per unit volume), and they produce lower levels of CO and CO_2 _emissions per kilometre travelled. However, diesel engines generally produce higher levels of particulates (eg, PM_2.5 _and PM_10_), which is a concern from a public health perspective. Furthermore, diesel cars usually have higher NO_x_/CO_2 _ratios than for petrol cars with three-way catalytic converters. Then again the particulate problem can be mitigated with some modern diesel cars that have diesel particulate filters. Also some diesel cars also have catalytic converters in the exhaust which reduce CO and unburnt hydrocarbon emissions. Nevertheless, in the New Zealand setting the use of catalytic converters is rare for any type of car.

• Petrol-fuelled cars may compete more favourably with diesels if these cars use biofuels that improve the overall CO_2 _emissions profile (due to the absorption of CO_2 _by plants used to produce the fuel). However, this depends very much on the type of biofuel and for some the net greenhouse benefit may be marginal or even involve greater aggregate environmental costs than do fossil fuels [19].

• While diesel engines are more efficient than petrol engines, they may have higher levels of embodied energy. That is, more energy is generally consumed (and hence more CO_2 _emissions produced) in manufacturing the larger sized diesel engines.

### Limitations

This study was limited to a modest sample of print media containing light passenger vehicle advertisements (albeit the two major monthly current affairs magazines with a wide readership). Nevertheless, given the absence of other similar studies in New Zealand or elsewhere, it does provide some initial baseline information on this topic. Although the advertised cars were fairly similar to new cars sold in the New Zealand market, the performance features of the advertised cars are likely to be far better (ie, more efficient and less polluting) than older vehicles still in use. Indeed, it has been estimated that nearly 50% of the New Zealand car fleet is more than 10 years old, and only 20% is less than five years old [20]. Nevertheless, we selected this five-year time period of advertisements to represent current vehicle technologies, and how these were marketed during a period of increasing concern about both greenhouse gases and air pollution.

There was the limitation of the lack of emissions data for New Zealand vehicles from official websites (ie, only fuel efficiency data were available). Because the emissions data that we used was sourced from an Australian Government website, there was a possibility of some differences with New Zealand vehicles though in general the types of car fleets of both countries appear to be fairly similar.

One issue not explored by this article is how some vehicle advertising may also promote driving behaviour that exacerbates the production of pollutants. We have described elsewhere that this same set of advertisements included in this study frequently contained imagery of speed, power references or acceleration data [8]. It is well-established that high speeds and high levels of acceleration lead to higher levels of emissions. Another limitation may relate to the validity of the ratings used. For example, one study in *Consumer Reports *found that around 90% of vehicles tested failed to achieve the stated US Environmental Protection Agency fuel efficiency ratings [21].

To address some of these limitations, future research could expand the sampling of advertisements to a wider range of magazines, newspapers and to televised car advertisements. It could also collect more denominator background on trends in expenditure on vehicle advertising in different media. As the typical fuel efficiency of new vehicles advertised in New Zealand appears to be very different from European cars, there is a need for such research in a wide range of different countries.

### Possible implications for policy makers

Further research on vehicle advertising and emissions is highly desirable, and could be combined with further analysis of advertising and vehicle safety issues [1-5,8]. Nevertheless, given the combined importance of climate change, energy security and the substantive health impact from localised air pollution in many countries, there is an immediate need for improvements in the content of vehicle advertising. Regulation of advertising content may also be justified on consumer rights grounds alone. Indeed, the European Union (EU) already has requirements for the provision of information on vehicles' fuel consumption and CO_2 _emissions [22]. Such information is required in promotional material ("advertisements in newspapers, posters, brochures") used in marketing new cars.

To ensure maximal consumer reach, it would be desirable that governments demand even more than the EU system, with stronger requirements for advertising, including graphic symbols measuring fuel efficiency, greenhouse ratings and air pollution ratings. Currently, some countries have regulations that require graphic symbols for energy efficiency ratings on whiteware appliances (eg, New Zealand and Australia), along with other information requirements in the marketing of many other consumer goods (eg, pharmaceuticals, tobacco, processed food etc). Indeed, graphic picture warnings are now mandatory on all tobacco packaging in a number of countries.

## Conclusion

Most of the light passenger vehicle advertisements in the magazines in this study were not informative in terms of greenhouse gas emissions and other air pollutants. To address these issues, governments should regulate the content of such advertisements, as is already being done by the European Union and with the marketing of many other consumer products such as tobacco, pharmaceuticals and appliances.

## Competing interests

The authors declare that they have no competing interests.

## Authors' contributions

Three of the authors contributed to the protocol (NW, AM, GT). AM undertook the content coding and both AM and NW undertook analyses. All authors contributed to revising drafts and preparing the final manuscript.
